# Advanced organ protection strategies in aortic arch surgery: A narrative review

**DOI:** 10.1177/02676591261466148

**Published:** 2026-07-11

**Authors:** Theresia Feline Husen, Annesimone Lotfalla, Rick Schreurs, Erik Körver, Sander Verheule, Leon Schurgers, Michael Jacobs, Roberto Lorusso, Samuel Heuts, Elham Bidar

**Affiliations:** 1 Department of Cardiothoracic Surgery, Heart and Vascular Centre, 199236 Maastricht University Medical Centre, Maastricht, Netherlands; 2 Faculty of Medicine, 95338 University of Indonesia, Jakarta, Indonesia; 3 Cardiovascular Research Institute Maastricht (CARIM), 118066 Maastricht University, Maastricht, Netherlands; 4 Department of Vascular Surgery, Heart and Vascular Centre, 199236 Maastricht University Medical Centre, Maastricht, Netherlands

**Keywords:** aortic arch, cerebral perfusion, neuroprotection, myocardial perfusion, selective organ perfusion, cardiothoracic surgery

## Abstract

**Objective:**

Aortic arch surgery remains a complex procedure associated with significant morbidity and mortality. This review aims to provide insights into the current concepts and evidence regarding organ protection during this procedure.

**Methods:**

A non-systematic approach was used to identify relevant literature across three databases. Inclusion criteria comprised case reports/series, observational cohorts, or randomized controlled trials investigating perfusion strategies and other factors that influence clinical outcomes in aortic arch surgery, as well as experimental studies and key reviews pertinent to the topic.

**Results:**

Although hypothermic circulatory arrest (HCA) remains the current gold standard, interest in using higher temperatures in aortic arch surgery is increasing. To enable this, cerebral, myocardial, and visceral protection remain crucial, prompting the development of several strategies to facilitate higher-temperature surgery. A growing body of predominantly observational, single-center evidence has explored warmer, continuous multi-organ perfusion strategies. These emerging approaches have been associated with favorable outcomes in selected cohorts but have not been validated against the guideline-concordant standard.

**Conclusion:**

Advancements in cardiopulmonary bypass and perfusion techniques have driven an evolving interest in continuous normothermic organ perfusion as an emerging strategy rather than an established alternative to HCA in selected cases. Continuous normothermic multi-organ perfusion represents an intriguing and potentially important direction, but its adoption should remain selective until comparative clinical evidence becomes stronger.

## Graphical abstract

**Graphical Abstract:**
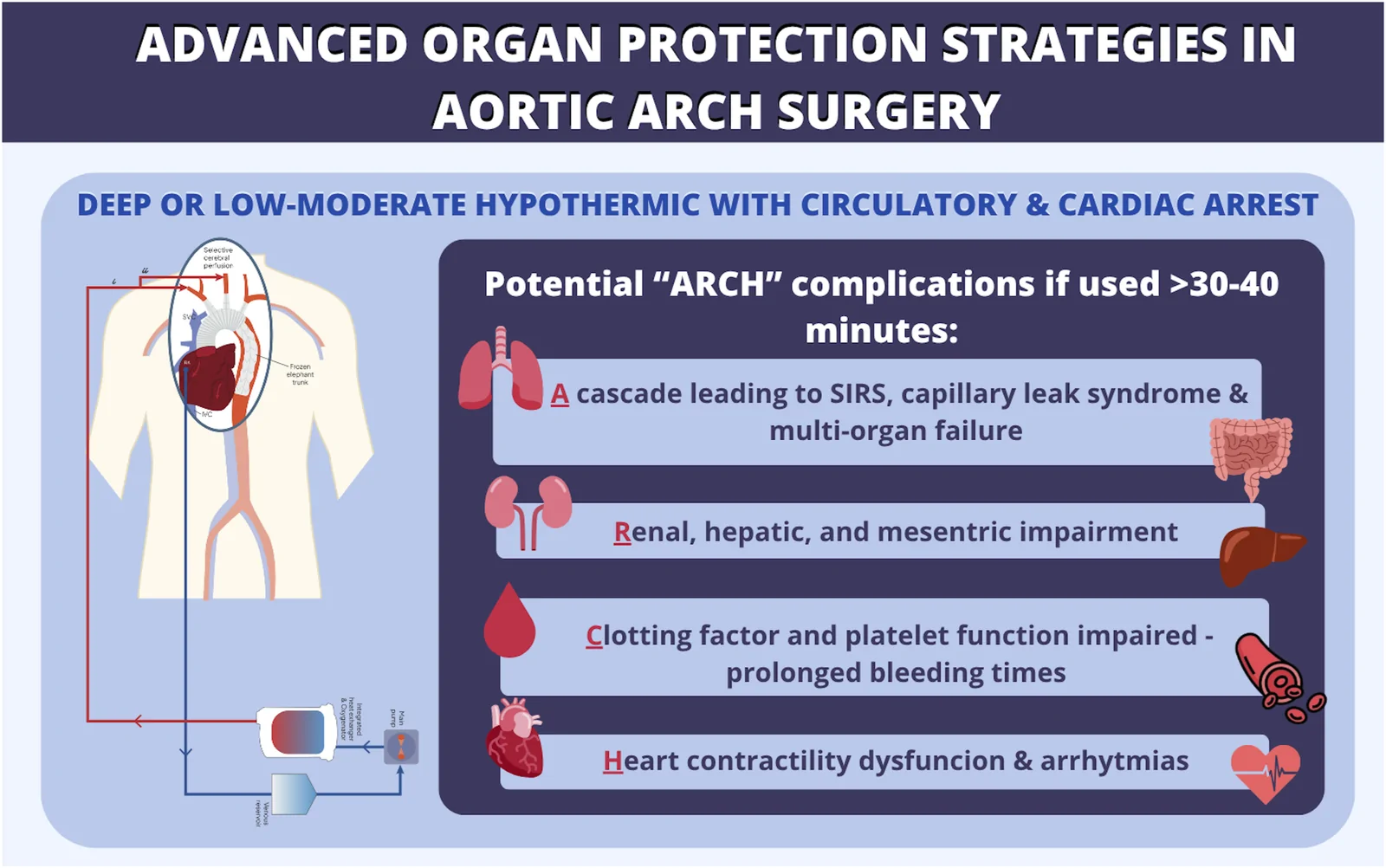

## Introduction

Although the field of thoracic endovascular aortic aneurysm repair is expanding, open aortic arch surgery remains the gold standard for the management of aortic arch pathology. These high-risk procedures are frequently undertaken as prophylactic interventions to prevent future dissection or aneurysm rupture, whereas acute aortic dissection mandates emergent intervention. Furthermore, these patients commonly present with significant comorbidities, often attributable to extensive atherosclerosis, placing them among the highest-risk groups in cardiovascular surgery, with a correspondingly elevated baseline risk of morbidity and mortality.^1^

The aortic arch lies at the crossroads of the heart, brain, and visceral organs, rendering surgery in this region both complex and high-risk, particularly with respect to neurological injury. Strategies for organ protection have consequently evolved considerably. Historically, aortic arch repair has relied upon deep hypothermic circulatory arrest (DHCA) in its purest form: a complete cessation of all organ perfusion, including that of the brain.^2,3^ Although this historical technique, which we hereafter denote as DHCA, perfused no organ at all, most centers now sustain the brain throughout the arrest period with continuous antegrade cerebral perfusion (ACP), such that arrest in contemporary practice increasingly applies to the lower body alone.^3^ In parallel, the degree of hypothermia employed has become more nuanced: the most recent EACTS/STS aortic guidelines stratify it into deep, low-moderate, high-moderate, and mild categories (Table 1). These classifications are typically based on nasopharyngeal, bladder, or rectal temperature measurements, although many centers utilize alternative sites, such as esophageal probes, which are not physiologically interchangeable and may therefore introduce discrepancies in the reported depths of cooling and rewarming across studies.^1^

**Table 1.** Contemporary hypothermia classification.^1^

Hypothermia category	Temperature
Deep	≤20°C
Low-moderate	20.1–24°C
High-moderate	24.1–28°C
Mild	>28°C

For myocardial protection, cardioplegic arrest is generally employed when concomitant replacement of the aortic root or proximal ascending aorta is required. Despite these advances, conventional approaches continue to carry substantial rates of postoperative neurologic and systemic complications.^2^

A recent meta-analysis of observational studies and randomized trials reported an association between warmer perfusion strategies and favorable clinical outcomes in selected populations.^4^ Additionally, several studies have reported comparable or potentially superior results with techniques that avoid cardioplegic arrest.^5,6^ Whereas previous reviews have largely addressed cerebral protection or temperature strategy in isolation, this narrative review integrates cerebral, myocardial, and visceral protection within a unified framework, contrasting the emerging concept of continuous, warmer multi-organ perfusion with the current guideline-concordant standard. In doing so, it examines the foundations of both conventional and emerging strategies in multi-organ protection during aortic arch surgery, with particular emphasis on the potential of physiological perfusion techniques.

## Methods

This review primarily explores perfusion strategies for aortic arch surgery, together with additional surgical factors that may influence clinical outcomes. A non-systematic, theme-driven approach was adopted to identify relevant literature. Three electronic databases were searched: Google Scholar, MEDLINE, and Scopus. The final search was conducted on February 15, 2025, with no lower date limit applied. Search terms included “cerebral perfusion,” “myocardial perfusion,” “beating heart,” “non-cardioplegic arrest,” “visceral perfusion,” “circulatory arrest,” “temperature,” “hypothermic,” “lower body perfusion,” “surgical techniques,” “aortic occlusion devices,” “surgical biomarkers,” “clinical outcomes,” and “arch surgery,” combined with relevant synonyms and Boolean operators where applicable.

Studies considered for inclusion comprised randomized controlled trials (RCTs), observational cohorts (both retrospective and prospective), and case reports and series investigating perfusion strategies and other factors affecting clinical outcomes in aortic arch surgery. Experimental studies, key review articles, and guidelines with their references pertinent to the topic were also included. To mitigate selection bias, studies were screened and prioritized according to their level of evidence: RCTs and large-scale observational cohorts were given precedence in the evaluation of established clinical practices, whereas case reports, case series, and experimental studies were used to identify emerging physiological trends and exploratory signals. Review articles and guidelines served to contextualize current clinical standards and to provide a comprehensive framework for multi-organ protection strategies. Conflicting data across studies were addressed by comparing study populations, procedural complexity, and the rigor of outcome reporting. Where randomized data, observational data, and physiologic reasoning diverged, the discrepancy is described explicitly in the relevant section, with randomized data taking precedence for general practice recommendations and observational data reserved for hypothesis generation.

The full text of the selected articles was reviewed to extract information on perfusion strategies and other surgical factors influencing clinical outcomes in aortic arch surgery. Key themes – systemic, myocardial, and cerebral perfusion – were synthesized into a cohesive framework to illustrate their respective effects, interactions, and management strategies.

## Results

Organ protection in aortic arch surgery is best understood as a single, interdependent problem rather than a series of separate techniques. Cerebral, myocardial, and visceral protection are not pursued in isolation: a strategy chosen to safeguard one organ system inevitably shapes the risk profile of the others, as well as the monitoring and cannulation required to support them. The degree of hypothermia, the choice between circulatory arrest and continuous perfusion, the route and laterality of cannulation, and the intensity of intraoperative monitoring are therefore interdependent decisions, each of which redistributes risk across the cerebral, myocardial, and visceral domains simultaneously. The sections that follow examine these elements in turn, but each is best read as one facet of this shared framework, which is brought together in the combined and beating-heart strategies in the final section.

This framework, in turn, is not applied uniformly across cases. Aortic arch surgery spans a heterogeneous range of procedures and patient contexts—from elective hemiarch replacement, through complex total arch and frozen elephant trunk repair, to acute type A dissection, reoperative surgery, and high-risk patients with limited physiological reserve—and the appropriate temperature target, arrest duration, myocardial ischemic time, spinal-cord protection, cerebral perfusion strategy, and cannulation route differ accordingly. The evidence discussed below should therefore be read in relation to the procedure and patient context in which it was generated, a distinction developed in detail in Section 3.6.

### Hypothermic circulatory arrest

Conventional aortic arch surgery has historically employed DHCA to provide a bloodless surgical field and an unobstructed operative view.^7^ Hypothermia protects the organs by reducing cellular metabolic rate, oxygen consumption (V̇O_2_), and the inflammatory response. Notably, a 10°C reduction in core temperature lowers V̇O_2_ and carbon dioxide production (V̇CO_2_) to approximately 50% of normothermic values.^8^ Additionally, lower temperatures increase the solubility of O_2_ and CO_2_ in blood and tissues, thereby reducing the partial pressures of these gases.^8^ To address these changes, α-stat management is generally preferred over the pH-stat strategy, as it preserves cerebral autoregulation and is associated with more favorable long-term neurological outcomes.^9^

These mechanisms are further informed by the Deep hypothermia and Retrograde cerebral perfusion Against Moderate hypothermia and Antegrade cerebral perfusion for Aortic arch replacement (DRAMA) trial.^10^ This randomized pilot study demonstrated that 100% of patients in the warmer moderate HCA (MHCA) + ACP group (26.3°C) developed new postoperative diffusion-weighted MRI (DWI) lesions, compared with 45% in the DHCA + retrograde cerebral perfusion (RCP) group (19.9°C); the MHCA + ACP group also exhibited a significantly higher mean number of lesions.

These findings introduce an important nuance into the broader trend toward warmer hypothermia. The increased embolic burden in the MHCA + ACP arm was attributed primarily to the additional manipulation and clamping of the innominate and left common carotid arteries required for ACP cannulation; conversely, the retrograde perfusion direction used in the DHCA + RCP arm may have conferred an embolic washout effect during the brief arrest period. Although the new DWI lesions did not translate into significant differences in the 6-month global neurocognitive score, the trial was not powered for clinical neurocognitive endpoints, and the high lesion incidence suggests a subclinical neurological vulnerability that must be weighed against the systemic benefits of moderate hypothermia. Importantly, the pre-specified short arrest duration in the trial (<30 min) means that its conclusions do not necessarily generalize to extended total arch repair or frozen elephant trunk procedures, in which moderate hypothermia combined with ACP retains a strong rationale on the basis of cumulative ischemic risk. Taken together, DRAMA supports a strategy-aware rather than a temperature-only interpretation of the shift toward moderate hypothermia: the systemic benefits of warmer perfusion are best realized when accompanied by careful attention to cerebral cannulation, supra-aortic vessel manipulation, and intra-operative embolic monitoring.^10^

In a secondary analysis of a separate trial, the Cognitive Effects of Body Temperature During Hypothermic Circulatory Arrest (GOT ICE) study found that the DHCA group outperformed the high and the low-moderate group in structured verbal memory, despite comparable global cognitive changes.^11^

Hypothermia may, however, lead to intracellular calcium accumulation, which is further exacerbated by ischemia-reperfusion injury (IRI) during the rewarming and reperfusion phases.^8^ This may result in (ventricular) arrhythmias, impaired myocardial contractility, and an increased risk of larger ischemic areas.^12^ Rewarming further compounds IRI, compromising mucosal and endothelial barriers and increasing their permeability.^8,12^

Systemically, the use of cardiopulmonary bypass, particularly in combination with multiple transfusions, may trigger systemic inflammatory response syndrome (SIRS).^13^ IRI also produces SIRS-like manifestations and may precipitate capillary leak syndrome. This systemic inflammation drives capillary leak, resulting in significant albumin loss from the intravascular space, which necessitates volume resuscitation and may culminate in multi-organ failure, with consequently high rates of morbidity and mortality.^12,13^

HCA also impairs the coagulation cascade, prolonging bleeding times.^8^ The resulting progressive hypovolemia warrants further transfusion, thereby exacerbating SIRS (Figure 1).^13^ Although HCA provides effective organ protection within 30–40 min, its associated adverse effects may disproportionately affect patients of advanced age and those with significant comorbidities.^1,14^ The GOT ICE trial reported significant reductions in cerebral gray matter volume, cortical thickness, and regional brain functional connectivity across all HCA depths.^11^ Because both the GOT ICE and DRAMA trials applied HCA for less than 30 min, their findings may not be generalizable to all arch procedures (Table 2). The extent of the repair and the duration of HCA must therefore be considered when selecting a temperature strategy for an individual patient.^11,15^

**Figure 1.**
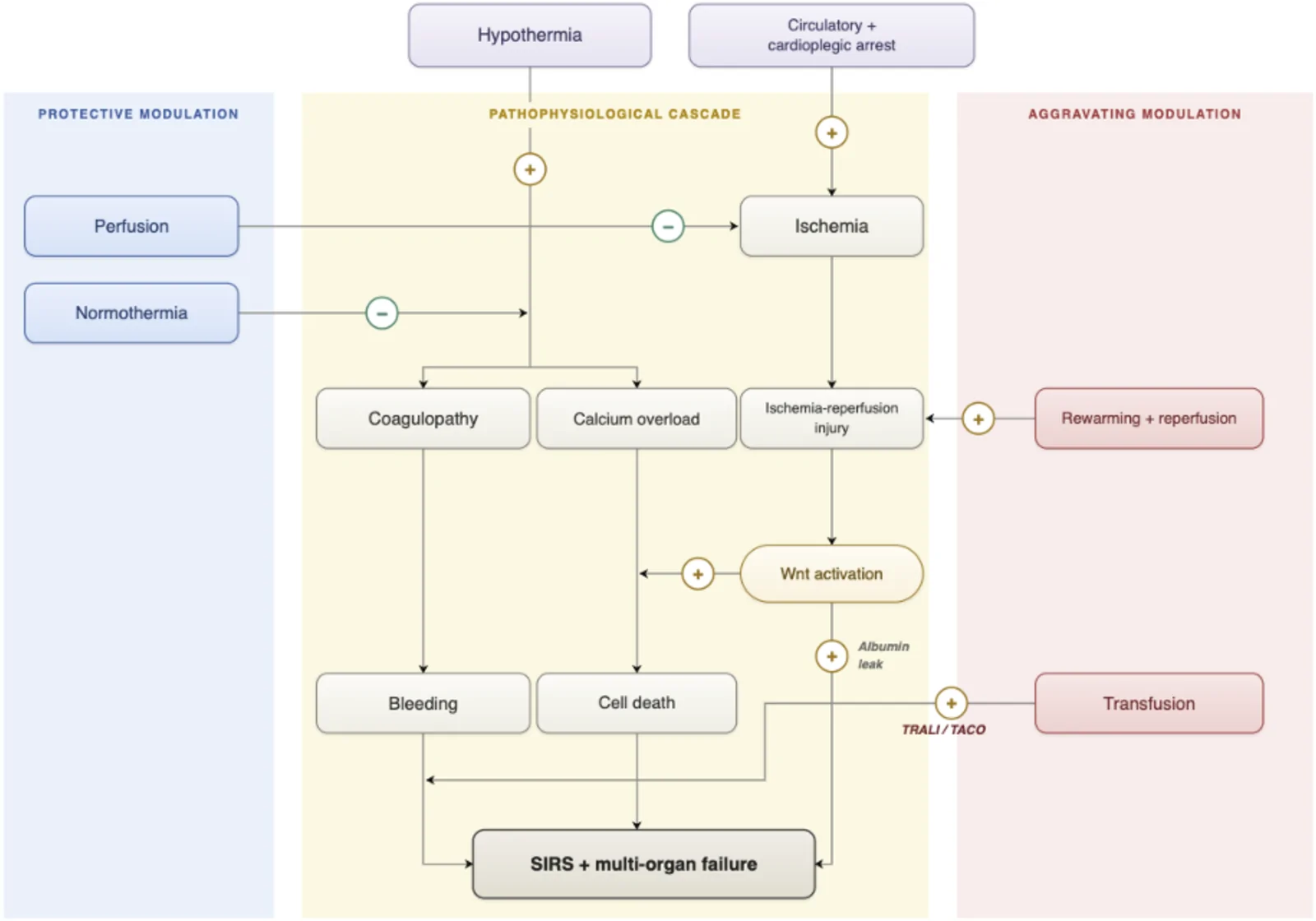
Pathophysiology of systemic inflammatory response syndrome (SIRS) during aortic arch surgery. This schematic outlines the systemic inflammatory response and cellular stress induced by hypothermic circulatory arrest, ischemia-reperfusion injury, and cardiopulmonary bypass.^8,12,13^ SIRS: systemic inflammatory response syndrome.

**Table 2.** Deep hypothermic circulatory arrest in aortic arch surgery patients.

Category	Advantages	Disadvantages	Study type	Level of evidence
Metabolism & pH	Reduces V˙O2 and V˙CO2 by ∼50% per 10°C drop; stabilizes membranes via an alkaline pH shift.	Induces metabolic acidosis and lactate accumulation; an alkaline shift can impair enzyme function and oxygen delivery.	Review	C
Cellular stability	Slows injury-related enzymatic activity, reduces metabolism, providing protection during short ischemic windows (<30–40 min).	Triggers calcium overload and cellular edema due to ATP-dependent ion channel/SERCA failure.	Review and expert consensus	C
Setup & logistics	Standardized and easy; requires no additional CPB cannulas, pumps, or complex lines.	Time-constrained; short safe window (<30–40 min) lacks generalizability for complex or extensive arch repairs.	Expert consensus and clinical guideline	C
Surgical field	Provides an unimpaired, bloodless view without additional perfusion catheters in the way.		Expert consensus	C
Systemic impact	Initial reduction in SIRS during the 3–40 min arrest period.	• Triggers SIRS and IRI; Wnt-mediated pathways exacerbate mitochondrial dysfunction, oxidative stress, arrhythmias, cardiac contractility dysfunction, capillary leak, multi-organ failure, and morbidity. • Impairs hemostasis; cold-induced platelet and clotting factor dysfunction leads to prolonged bleeding.	Review and cohort retrospective	C

The current EACTS/STS aortic guidelines likewise recommend that the choice of temperature be guided by the anticipated extent of repair, the expected HCA duration, and the presence of preoperative malperfusion (recommendation class IIa, level of evidence C, without references).^1^ Accordingly, more extensive repair, longer HCA time, and the presence of malperfusion favor a lesser degree of hypothermia. These guidelines further recommend high-moderate lower body HCA in combination with ACP for complex procedures.^1^ Nevertheless, considerable discrepancy persists in temperature management during HCA between centers, ranging from 18°C to 30°C,^16,17^ underscoring the need for further research.

### Lower-body perfusion

Retrospective cohort studies have reported that continuous lower-body perfusion at warmer temperatures (e.g., moderate HCA or normothermia, 36-37°C) may promote early serum lactate clearance, normalization of acidosis, and metabolic recovery. These physiological benefits have been associated with favorable clinical outcomes in selected observational, non-randomized cohorts,^18^ including lower rates of mortality and stroke.^16^ These advantages, however, are contingent on continuous flow: because higher temperatures increase metabolic demand, making the brain and spinal cord correspondingly more vulnerable to ischemic injury should perfusion be interrupted. Effective spinal cord protection, therefore, rests on three measures. First, collateral perfusion is preserved through mild-to-moderate HCA combined with ACP. Second, a mean arterial pressure (MAP) of approximately 80–100 mmHg is maintained. Third, cerebrospinal fluid drainage is employed. These measures are ideally guided by patient-specific risk stratification.^19^

Consequently, retrospective cohorts and expert consensus indicate that warmer perfusion strategies require tight control of perfusion flow and pressure.^3,20^ Several studies have therefore investigated continuous distal (lower-body) perfusion combined with concurrent cerebral perfusion, an approach that avoids HCA to both the brain and the visceral organs. This is typically achieved by occluding the descending aorta and perfusing the distal aorta, and has been associated with potential benefits, including preserved urine output, hepatic function, and overall tissue perfusion, thereby potentially reducing postoperative multi-organ failure. Such outcomes can be monitored using markers such as creatinine, cystatin C, and liver enzymes.^20,21^ Table 3 presents the various reported surgical techniques for lower-body perfusion that avoid HCA.

**Table 3.** Lower-body perfusion in total aortic arch replacement.

Parameters	Techniques options^a^									
	Studies	Available surgical techniques^1,2^	Conventional^1,2^	Current trend^1,2^	Touati et al. (2007)^20^	Bachet et al. (1991)^24^	Abu-omar et al. (2013)^26^	Di Natale et al. (2013)^25^	Martens et al. (2015)^5^	MUMC+^44,45^
	Study type (level of evidence)	Expert consensus & reviews (C)	Expert consensus & reviews (C)	Expert consensus & reviews (C)	Cohort retrospective (C)	Case series (C)	Case report (C)	Case report (C)	Cohort retrospective (C)	Cohort retrospective (C)
	Total sample	-	-	-	29	54	1	1	144	121
Temperature		1. ≤20°C 2. 20.1–24°C 3. 24.1–28°C 4. 28.1–36°C 5. Normothermia (36–37°C)	1. ≤20°C 2. 20.1–24°C	20.1–28°C	36°C–37°C	6–12°C	20°C	36°C	25°C	28–32°C
Circulatory arrest		With or without	With	With	Without	With	Without	Without	With	Without
Aortic occlusion device (during lower body perfusion)		If without circulatory arrest:	-	-	Robicsek Pruitt aortic occlusion catheter or clamp in descending aorta, aortic clamps in supraaortic vessels (innominate, left common carotid, and left subclavian artery)	-	Aortic clamp in the ascending and descending aorta	Endoclamp aortic catheter in the descending aorta and aortic clamp in the ascending aorta	Aortic clamp on ascending aortic prosthesis after root completion with a CMP line. Lower body perfusion through the side arm of the arch prosthesis, with clamping of the prosthesis	Coda Balloon catheter in the descending aorta
		Aortic clamp or aortic occlusion balloon in the descending aorta					Inflated balloon catheter in the innominate and left common carotid artery			

CMP: continuous myocardial perfusion; MUMC: Maastricht University Medical Center+.

^a^*Note.* The “Conventional” and “Current trend” columns reflect guideline-concordant standard practice; the individual study columns and the MUMC + column represent examples of reported practice rather than evidence-based recommendations.

Maintaining adequate flow and pressure is equally important for preventing ischemia on the one hand and tissue damage from overperfusion on the other, the latter of which can trigger inflammation and oxidative stress. Animal data suggest that optimal perfusion parameters vary by organ and temperature. In porcine models, for example, lower body perfusion at a flow rate of 20 ml/kg/min with a pressure exceeding 40 mmHg preserved approximately 50% of mesenteric flow and reduced systemic stress compared to non-perfused conditions.^22^ These findings underscore the need for further research into optimal perfusion parameters at warmer temperatures.

### Myocardial perfusion

In open aortic arch surgery, most centers still employ cardiac arrest with antegrade or retrograde cardioplegia (crystalloid or blood), administered as a single dose, intermittently, or continuously. Evidence from a retrospective cohort study suggests, however, that in patients with pre-existing impaired cardiac function, cardioplegia may be ineffective or even deleterious.^5^ In aortic arch surgery, cardioplegic arrest times of 2.5–3.5 h are frequently encountered, even at experienced high-volume institutions, as reported in a recent two-center retrospective study.^23^ Such prolonged ischemic intervals necessitate meticulous strategies for myocardial protection throughout the procedure. Within the conventional cardioplegic paradigm, the 2024 EACTS/STS guidelines accordingly recommend retrograde cardioplegia to permit repeat doses without interrupting the procedure (class IIa, level C).^1^The limitations of prolonged cardioplegic arrest have nonetheless prompted interest in approaches that depart from cardioplegia altogether.

Since the late 1990s, continuous myocardial perfusion (CMP) with cold blood has been described as a non-cardioplegic alternative, in which the myocardium is perfused throughout arch repair rather than arrested.^24^ CMP has been employed predominantly in pediatric patients, in whom beating-heart arch repair – typically combined with ACP – has been used to minimize myocardial ischemia and has proved technically feasible and safe.^6^ More recently, several centers have extended continuous, non-cardioplegic myocardial perfusion to high-risk adult cases, reporting their experience in retrospective case reports and case series.^5,25,26^

A retrospective study by Martens et al.^5^ investigated cold-blood CMP in adults undergoing complex repairs, with or without concomitant aortic root procedures; the details of these techniques are summarized in Table 4. During HCA and arch repair, the heart was continuously perfused with cold blood via a root cannula, substantially reducing cardioplegic arrest times. Although ventricular fibrillation occurred frequently during the procedure, CMP adequately met myocardial energy demands, as evidenced by the absence of any association between ventricular fibrillation and adverse cardiac outcomes in the CMP group. CMP patients also demonstrated significantly lower postoperative lactate levels over 48 h, together with a trend toward lower creatine kinase-MB levels that did not reach statistical significance. Clinically, these findings translated into a significantly lower incidence of low cardiac output syndrome, reduced bleeding, and lower mortality.^5^ These results suggest that CMP may represent a more favorable approach to myocardial protection, particularly in high-risk patients, although they derive from a single-center retrospective cohort and require prospective validation before broader adoption. Consistent with these findings, the latest EACTS/STS guidelines state that non-cardioplegic myocardial perfusion may be considered during extended aortic arch repair to reduce myocardial ischemia time (recommendation level IIa, level of evidence C, without references).^1^ Literature findings on myocardial protection during arch surgery are summarized in Table 4.

**Table 4.** Myocardial perfusion in total aortic arch replacement.

Parameters	Techniques options^a^									
	Studies	Available surgical techniques^46–49^	Conventional^46–49^	Current trend^46–49^	Touati et al. (2007)^20^	Bachet et al. (1991)^24^	Abu-omar et al. (2013)^26^	Di Natale et al. (2013)^25^	Martens et al. (2015)^5^	MUMC+^44,45^
	Study type (level of evidence)	Expert consensus & reviews (C)	Expert consensus & reviews (C)	Expert consensus & reviews (C)	Cohort retrospective (C)	Case series (C)	Case report(C)	Case report (C)	Cohort retrospective (C)	Cohort retrospective (C)
	Total sample	-	-	-	29	54	1	1	144	121
Continuity		1. Continuous	None	None	Intermittent (repeated every 7–10 min) (36°C–37°C)	Continuous	Continuous	Continuous	Continuous	Continuous
		2. Intermittent								
		3. None								
Flow direction		1. Antegrade	None	None	Retrograde	Antegrade	Antegrade	Antegrade	Retrograde	Antegrade
		2. Retrograde								
		3. Combined								
		4. None								
Flow amount (ml/min)		>250 ml/min			N/A	Total blood flow: 400-500	Total blood and cardiac flow: 250	Total blood flow: 800	Total blood flow: 150–500	300-500
						Cardiac flow: 150			Cardiac flow: ±350	Also based on potential ECG deviations (ischemia or dilation of the ventricle)
Pressure (mmHg)		30–50			30–40	N/A	N/A	N/A	80–100	70–90
Cardioplegic arrest		With or without	With	With	With	Without (perfused using cold blood [6–12°C])	Without (perfused using warm blood (34°C))	Without (perfused using warm blood (36°C))	Without (perfused using cold blood (25°C))	Without (perfused using warm blood (36°C))
Cardioplegia type		1. Crystalloid	Cold crystalloid or blood	Warm blood	Warm blood	None	None	None	None	None
		2. Blood								
		3. Mixture								
Cardioplegic temperature		1. Cold cardioplegia: 4–15°C 2. Intermediate lukewarm (Mixture between warm & cold, varies between centers) 3. Warm cardioplegia: 34–37°C	Cold cardioplegia: 4-15°C	1. Cold cardioplegia:4-15°C 2. Warm cardioplegia: 34-37°C	Warm cardioplegia: 36-37°C	None	None	None	None	None
Cardioplegic flow direction		1. Antegrade	Antegrade	Antegrade	Retrograde	None	None	None	None	None
		2. Retrograde								
		3. Combined								
		4. None								

^a^*Note.* The “Conventional” and “Current trend” columns reflect guideline-concordant standard practice; the individual study columns and the MUMC + column represent examples of reported practice rather than evidence-based recommendations.

### Cerebral perfusion

Although deep hypothermia has traditionally been applied for its neuroprotective effects, it nonetheless carries a risk of ischemia and neurological deficits. A major advance in brain protection during aortic arch surgery has been the adoption of cerebral perfusion (CP), which most centers now employ. This CP encompasses ACP and RCP approaches, used alone or in combination. Retrospective studies have shown that CP reduces neurological complications and intubation time while maintaining comparable mortality rates.^3,27^ Moreover, when systemic temperatures are raised, the addition of CP has been associated with at least comparable, and in some cohorts, more favorable, clinical outcomes in observational data.^16,18^

Comparing the two approaches directly, a network meta-analysis of cohort studies and RCTs reported similar clinical outcomes between ACP and RCP, and this equivalence held across the range of temperatures studied.^28^ Cohort studies, meta-analyses, and expert consensus nonetheless indicate that ACP more closely mimics physiological flow and is more widely used than RCP. ACP can be performed unilaterally (uACP), bilaterally (bACP), or trilaterally (additionally perfusing the left subclavian artery). Bilateral or trilateral ACP is preferred in patients with an incomplete Circle of Willis. Trilateral perfusion is particularly important when the posterior cerebral circulation is dominated by the left vertebral artery, especially where the right vertebral artery is hypoplastic and terminates in the posterior inferior cerebellar artery. In patients with compromised collateral circulation or vertebral dominance, uACP may result in hypoperfusion, particularly when HCA time exceeds 30–40 min, although collateral flow through the external carotid artery provides some compensation.^1,3,29,30^ The EACTS/STS guidelines recommend ACP (class IIa, evidence B) and, in patients with an incomplete circle of Willis, a trilateral approach (class IIa, evidence C), with preoperative evaluation of cerebral anatomy.^1^ A retrospective observational study reported that intraoperative transcranial doppler (TCD) can monitor flow and pressure in real time, although technical limitations and patient-specific factors, such as poor acoustic windows or small vessel caliber, leave the true collateral potential uncertain; hypoperfusion or ischemic injury may still occur, particularly when HCA times are prolonged.^31^

RCP, in which blood is delivered retrogradely via the superior vena cava, has regained interest for its potential embolic washout effect and its utility when arterial cannulation is challenging.^32^ This lower embolic risk may be advantageous, given that a retrospective study by Berger et al.^33^ found stroke during arch surgery to be caused predominantly by embolization. However, prior meta-analyses and observational studies indicate that RCP is generally less effective for HCA durations >40 min and may increase cerebral edema through elevated venous pressure, although the evidence is inconsistent.^34,35^ Two-center retrospective data by Arnaoutakis et al. further demonstrated that RCP yields outcomes comparable to those of DHCA alone.^36^

Ultimately, brain protection depends on multiple factors, including perfusion time, temperature, flow, and pressure.^3^ A meta-analysis associated shorter cerebral perfusion times with reduced ischemic risk.^37^ Experimental studies suggest that cerebral perfusion MAP should be maintained between 50 and 90 mmHg to optimize neurological outcomes while preventing increased intracranial pressure (ICP) and edema.^38^ Observational studies recommend flow rates of 6–15 mL/kg/min, as higher flows may raise ICP and thereby increase the risk of cerebral edema and cerebral hyperperfusion syndrome.^3,20^ The various cerebral perfusion strategies are summarized in Table 5.

**Table 5.** Cerebral perfusion in total aortic arch replacement.

Parameters	Techniques options^a^									
	Studies	Available surgical techniques^3,20^	Conventional^3,20^	Current trend^3,20^	Touati et al. (2007)^20^	Bachet et al. (1991)^24^	Abu-omar et al. (2013)^26^	Di Natale et al. (2013)^25^	Martens et al. (2015)^5^	MUMC+^44,45^
	Study type (level of evidence)	Expert consensus & reviews (C)	Expert consensus & reviews (C)	Expert consensus & reviews (C)	Cohort retrospective (C)	Case series (C)	Case report (C)	Case report (C)	Cohort retrospective (C)	Cohort retrospective (C)
	Total sample	-	-	-	29	54	1	1	144	121
Flow direction		1. Deep hypothermic with total circulatory arrest	Deep hypothermia with total circulatory arrest	Bilateral antegrade	Unilateral antegrade	Unilateral antegrade	Bilateral antegrade	Unilateral antegrade	Unilateral antegrade	TCD- and EEG-guided, which determines the approach: Unilateral/bilateral/trilateral (antegrade)
		2. Unilateral or bilateral with antegrade or retrograde								
Perfusion flow		6–15 ml/min/kg of body weight (0.6–1.6 ml/min/g of cerebral tissue)		10–15 ml/min/kg of body weight	1–1.6 ml/min/g of cerebral tissue)	250–350 ml/min	N/A	N/A	10 ml/kg/min	10 ml/min/kg (in case of 1 cannula) or 7 ml/min/kg/cannula with cannulas
		(Varieties existed between centers & references)								
Pressure (mmHg)		50–70		60	70	60–70	50–60	N/A	50–60	Minimum of 50
										Also, based on measurements and guidance from the clinical neurophysiologist

^a^*Note.* The “Conventional” and “Current trend” columns reflect guideline-concordant standard practice; the individual study columns and the MUMC + column represent examples of reported practice rather than evidence-based recommendations.

### Cannulation and monitoring

Other factors that may influence surgical outcomes include the choice of cannulation site (Table 6). Arterial cannulation for aortic arch surgery follows either an antegrade (physiological) or retrograde flow direction. Antegrade sites include the ascending aorta, the arch, and the innominate, subclavian/axillary, or carotid arteries. Retrograde cannulation, typically via the femoral artery, is faster and useful in hemodynamically unstable patients, but carries a risk of malperfusion, embolization, or false-lumen perfusion in dissection. Antegrade perfusion is therefore preferred for arch replacement. The right axillary/subclavian artery is commonly used, as it enables ACP during clamping of the innominate artery, although cannulation is time-consuming and may cause local complications such as brachial plexus injury and seroma. Innominate artery cannulation has more recently emerged as a simpler, incision-sparing alternative that streamlines the establishment of ACP. The ACE CardioLink-3 trial found comparable neuroprotection between the two sites, albeit with a high burden of new MRI-detected ischemic lesions in both groups.^15^

**Table 6.** Other contributing factors in total aortic arch replacement.

Parameters	Techniques^a^									
	Studies	Available surgical techniques^2,3^	Conventional^2,3^	Current trend^2,3^	Touati et al. (2007)^20^	Bachet et al. (1991)^24^	Abu-omar et al. (2013)^26^	Di Natale et al. (2013)^25^	Martens et al. (2015)^5^	MUMC+^44,45^
	Study type (level of evidence)	Expert consensus & reviews (C)	Expert consensus & reviews (C)	Expert consensus & reviews (C)	Cohort retrospective (C)	Case series (C)	Case report (C)	Case report (C)	Cohort retrospective (C)	Cohort retrospective (C)
	Total sample	-	-	-	29	54	1	1	144	121
Arterial pressure (mmHg)		80–90			55–70	200–250	N/A	75	N/A	50–70
Aortic occlusion device		Aortic clamp or aortic occlusion balloon			Robicsek Pruitt aortic occlusion catheter and aortic clamp	Aortic clamp	Aortic clamp	Endoclamp aortic catheter and aortic clamp	Aortic clamp	Coda Balloon catheter
Location of CPB cannulation		Arterial line: Subclavian-axillary, carotid, femoral, or innominate artery, or ascending or descending aorta		Arterial line: Subclavian-axillary or carotid artery	Arterial: Brachiocephalic trunk, left common carotid artery, and right femoral artery.	Arterial line: Femoral artery and brachiocephalic trunk	Arterial line: Femoral artery, right and left carotid artery	Arterial line: Femoral artery and ascending aorta	Arterial line: Ascending aorta	TCD-guided; arterial line: Left and/or right axillary-subclavian artery, and/or left carotid artery, and femoral artery (+CMP)
		Venous line: Right atrium or bicaval cannulation		Venous line: Right atrium	Venous line: Right atrium	Venous line: Right atrium	Venous line: Right atrium and inferior vena cava	Venous line: Right atrium and inferior vena cava	Venous line: Right atrium or bicaval cannulation	Venous line: Right atrium

^a^*Note.* The “Conventional” and “Current trend” columns reflect guideline-concordant standard practice; the individual study columns and the MUMC + column represent examples of reported practice rather than evidence-based recommendations.

Aortic occlusion techniques using external clamps or endo-aortic balloons require caution in dissection owing to tissue fragility. By occluding the descending aorta and allowing earlier resumption of lower-body perfusion, balloon occlusion can markedly shorten the HCA time and raise the nadir hypothermic temperature, while achieving comparable rates of mortality and major adverse events such as stroke and pulmonary infection.^39^ (Table 6).

Beyond these technical considerations, intraoperative monitoring serves to verify the adequacy of perfusion and to enable real-time adjustment of temperature, flow, and pressure, and is most usefully understood as a layered approach spanning systemic, cerebral, and organ-specific domains. At the systemic level, glucose, hematocrit, coagulation status, and acid-base balance are routinely tracked. For cerebral monitoring, the available modalities include electroencephalogram (EEG), the bispectral index (BIS), somatosensory evoked potentials (SEPs), and oxygenation- and flow-based monitors such as transcranial doppler (TCD), jugular venous oxygen saturation, and near-infrared spectroscopy (NIRS). NIRS is valued intraoperatively for guiding cannulation and detecting acute malperfusion or high-risk states; its restricted frontal-lobe coverage, however, means that it correlates only weakly with postoperative neurological outcomes, so it is best regarded as a real-time safety monitor rather than a predictor of recovery.^40^ Accordingly, while many centers rely on NIRS and BIS, more advanced modalities such as EEG, SEPs, and TCD provide more detailed real-time data on cerebral perfusion.^40^ In warmer temperature perfusion strategy, more extensive monitoring is crucial as higher temperature increases metabolic demands causing heightened risk to the cerebral and spinal cord.^19^

At the organ-specific level, monitoring includes pulmonary artery catheters, TEE, and bilateral radial artery pressure monitoring, particularly during axillary cannulation; a distal femoral arterial line may further support comprehensive perfusion monitoring – relevant, for instance, to spinal cord injury (SCI) – alongside SEPs and somatic NIRS.^19,40,41^

### Variety of aortic arch procedures

As outlined in the preceding sections, maintaining adequate organ perfusion during HCA is of central importance in aortic arch surgery. The optimal combination of protection strategies cannot, however, be generalized, as aortic arch surgery encompasses a highly heterogeneous spectrum of procedures and pathologies. Target temperatures, arrest durations, and organ perfusion strategies are therefore not interchangeable across cases. Elective hemiarch replacement, for instance, generally requires shorter HCA times, permitting the safe use of moderate-to-mild hypothermia combined with ACP and standard cardioplegic arrest.^2,16^ More extensive procedures, such as total arch replacement (TAR) and frozen elephant trunk (FET) implantation, by contrast, entail considerably longer HCA and myocardial ischemic times. Deployment of the stent graft in FET procedures additionally raises the risk of SCI, often necessitating lower core temperatures, bilateral ACP, and adjunctive strategies such as CMP to mitigate cumulative ischemic injury.^5,41^

Acute type A aortic dissection (ATAAD) and high-risk redo cases present distinct physiological challenges that call for tailored approaches. ATAAD is a life-threatening emergency characterized by tissue fragility and malperfusion syndromes, in which cannulation choices and the selection between ACP and RCP must be individualized according to the unpredictable operative scenario and the risk of embolic debris.^35,42,43^ High-risk reoperative arch surgery is further complicated by severe adhesions and altered anatomy, which substantially prolong surgical dissection and HCA times.^2^ In such complex cases, deeper hypothermia is frequently employed to provide a broader safety margin, while non-cardioplegic CMP may be particularly valuable in preventing cumulative myocardial damage during a lengthy repair, and even in special populations, such as pediatric patients and adults with pre-existing impaired cardiac function.^5,6^ Weighing the anticipated HCA duration against the specific procedural risks is therefore an essential prerequisite before adopting any standardized combined perfusion protocol.

### Combining systemic, myocardial, and cerebral perfusion during aortic arch surgery: beating-heart arch surgery without circulatory arrest

In accordance with the 2024 EACTS/STS guidelines, most contemporary centres employ high-moderate HCA combined with ACP as the current clinical standard for complex arch repair. This guideline-concordant approach offers a well-characterized safety margin for arrest durations of approximately 30–40 min, with the temperature, cerebral perfusion strategy, and arrest duration individualized according to the anticipated extent of repair, the expected arrest time, and preoperative malperfusion status.

A growing body of predominantly observational evidence has explored more physiologic conditions, combining mild-to-normothermic systemic temperatures with continuous multi-organ perfusion – visceral, cerebral, and in some emerging techniques, myocardial perfusion without cardioplegic arrest. Several observational studies and case series of total aortic arch replacement have suggested that this approach may reduce the incidence of cardiac dysfunction, shorten intensive care unit and hospital stays, and lower the risk of multi-organ failure in selected patients.^5,20,24–26^ One observational study reported that CMP with warm blood under mild-to-moderate systemic hypothermia appeared to offer favourable organ protection and clinical outcomes.^16^ These observational signals must, however, be interpreted with caution: the supporting evidence consists predominantly of small, single-center, retrospective series; no randomized comparative data are currently available; and apparent benefits in selected cohorts cannot be considered evidence of superiority over the guideline-concordant standard. Moreover, warmer temperatures markedly increase metabolic demand and thereby heighten the risk of ischemic injury to vulnerable organs, notably the brain and spinal cord, should any interruption of flow occur.^19^ Maintaining continuous perfusion likewise requires meticulous control, since excessive cerebral blood flow or pressure can precipitate cerebral edema and hyperperfusion syndrome.^3,20^ It is precisely this dependence on uninterrupted flow that defines the rationale for continuous multi-organ perfusion: by sustaining perfusion throughout the repair rather than relying on circulatory arrest, the approach is intended to keep the warmer organs continuously supplied and thereby avoid the very interruption that would otherwise expose them to ischemic injury.

#### Emerging combined perfusion strategies: Institutional protocol

Building on this rationale, and to address the attendant need for stringent perfusion control, our institution has proposed a hypothesis-generating approach to advanced organ protection that combines cerebral, myocardial, and lower-body perfusion during elective and emergent aortic arch surgery. In the interest of transparency, we disclose that this protocol was developed by the authors’ own group, and the appraisal below is therefore presented with deliberate caution to avoid overstating its merits. This proposed approach combines continuous cerebral and lower-body perfusion under mild hypothermia with continuous myocardial perfusion using whole warm blood delivered via a separate pump and heat exchanger (Medtronic MYOtherm XP), providing normothermic (36°C) myocardial perfusion and thereby protecting against the prolonged cardioplegic arrest times and myocardial ischemia of conventional techniques. Under invasive aortic root pressure monitoring, flow is titrated – typically 300–500 mL/min to maintain an aortic root pressure of 70–90 mmHg – depending, among other factors, on the degree of aortic valve insufficiency and left ventricular unloading conditions. Further procedural details are published elsewhere.^44,45^ Figure 2 compares this proposed strategy with classical HCA.

**Figure 2.**
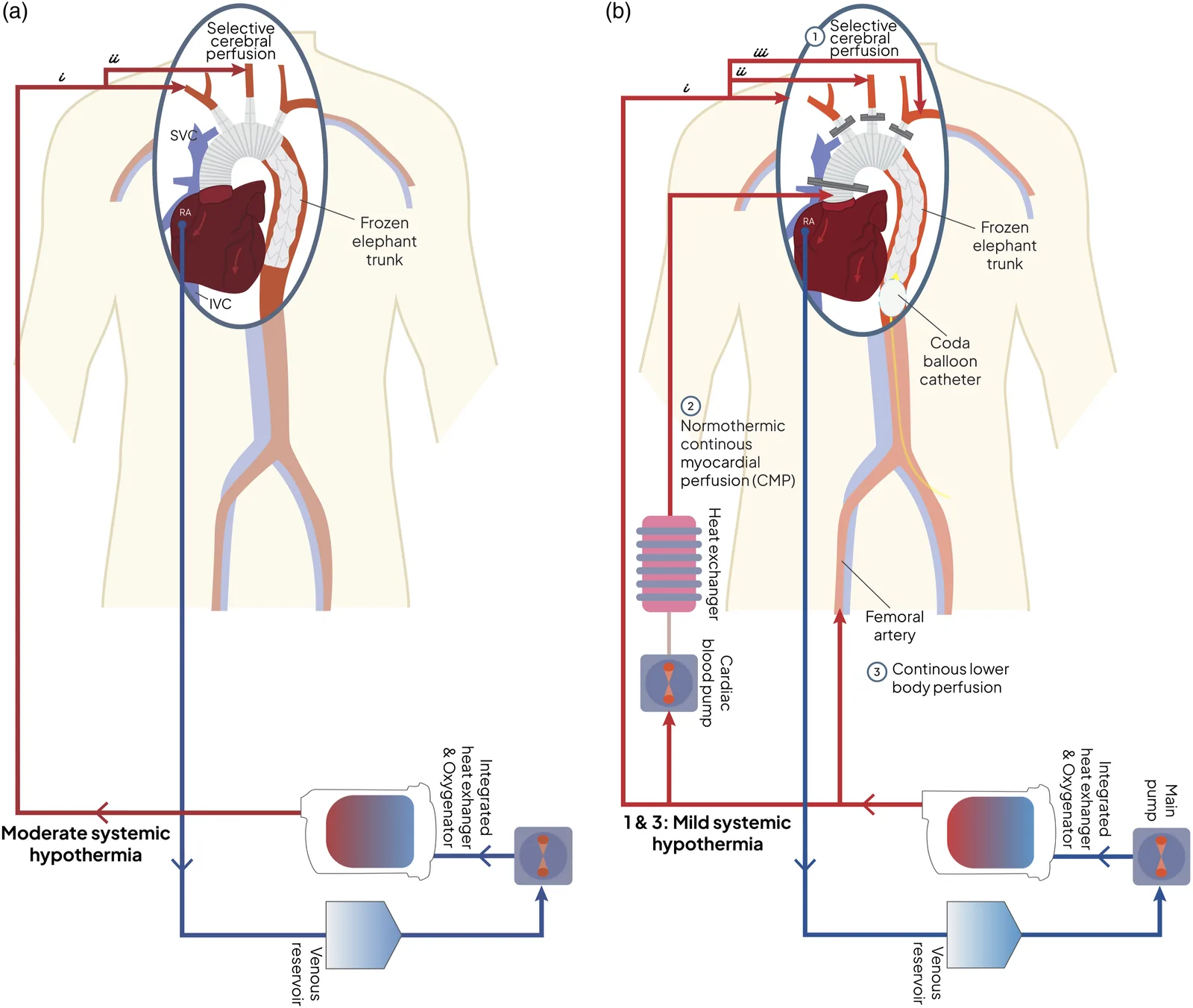
Perfusion strategies during aortic arch surgery. (a) Conventional hypothermic circulatory arrest (HCA) with selective antegrade cerebral perfusion unilateral (only i) or bilateral (i and ii); (b) Emerging integrated approach combining cerebral, myocardial, and lower-body perfusion (hypothesis-generating institutional protocol — see Section 3.7): Selective cerebral perfusion with normothermic continuous myocardial perfusion without circulatory arrest. Of note, in the case of complete arch replacement with implantation of the left subclavian artery, trilateral cerebral perfusion (i–iii) is achieved through indirect cannulation of the left subclavian artery using an 8 mm prosthesis. This panel depicts a novel institutional protocol developed at the authors’ own center (MUMC+). The current level of supporting evidence is limited to a single retrospective, single-center comparative analysis from the authors’ own group (ref. 45); no randomized comparative data are available. The approach should therefore be considered investigational, with selective adoption pending stronger comparative evidence.

While theoretically promising, this approach has not yet been tested against established gold-standard techniques in randomized comparisons, and its potential superiority and broader applicability require further evaluation. A first single-center retrospective comparative analysis from our group reported favorable biochemical and mortality outcomes relative to conventional techniques in elective aortic arch surgery; these findings are encouraging but must be interpreted with caution, given the retrospective, single-center design. Until stronger clinical evidence becomes available, adoption of this approach should remain tailored to the individual patient. In addition, several practical barriers and risks associated with this emerging multi-organ perfusion strategy must be acknowledged. Technologically, the approach introduces significant technical complexity, requiring additional cannulation sites and separate perfusion circuits, which increase the monitoring burden on the surgical and perfusion teams. The additional arterial cannulation sites required to establish combined cerebral, myocardial, and lower-body perfusion also broaden the surface for cannulation-related complications, raising the risk of embolization and malperfusion – including false-lumen perfusion – particularly in the fragile, dissected aorta, where these hazards are most pronounced. Furthermore, shifting toward warmer temperatures mandates an absolute dependence on uninterrupted flow; any accidental interruption drastically increases the risk of cerebral, spinal cord, or visceral ischemic injury due to the higher metabolic demands imposed by warmer temperatures. Surgeons must also remain cautious regarding the trade-off between hyperperfusion risks (e.g., cerebral edema) and hypoperfusion (e.g., cerebral ischemia) when applying these combined perfusion strategies, highlighting the critical importance of precisely managing perfusion flow and pressure. Finally, the reproducibility of this protocol outside highly experienced expert centers remains unproven, and a substantial learning curve is inherently required for its safe and widespread adoption.

## Conclusions and future directions

Aortic arch surgery remains a complex and high-risk intervention. The safest conclusion that can currently be drawn from the evolving spectrum of perfusion strategies is that continuous normothermic multi-organ perfusion represents an intriguing and potentially important evolving clinical concept rather than a universally established standard, one whose adoption should remain selective until comparative clinical evidence becomes stronger.

First, the current standard of care is individualized HCA with ACP, most commonly applied as high-moderate hypothermia for complex repair, with temperature, cerebral perfusion strategy, and arrest duration tailored to the extent of repair, the expected arrest time, and preoperative malperfusion status. Supported by randomized trials, large observational cohorts, and guideline consensus, this approach should remain the default for the majority of patients.

Second, warmer and continuous multi-organ perfusion strategies constitute an intriguing emerging direction that nonetheless remains unproven. The supporting evidence consists predominantly of single-center observational or institutional experience from pioneering centers, including preliminary comparative data from our own group, and any apparent benefits must be weighed against the absence of randomized comparative data and the increased procedural complexity.

Third, the principal knowledge gaps that must be addressed before broader adoption are now clearly identifiable: adequately powered randomized comparative trials with standardized clinical and neurocognitive endpoints; procedure-specific evidence across the heterogeneous spectrum of arch surgery; reproducibility data from beyond experienced single centers; and prospective characterization of the patient subgroups most likely to benefit.

Fourth, the adverse clinical outcomes after complex aortic arch surgery are highly multifactorial and cannot be attributed to a single pathophysiological pathway. Instead, they represent an intricate, multi-factorial interaction where hypothermia, circulatory arrest duration, cardiopulmonary bypass duration, mandatory blood transfusions, and rewarming-induced stress cross-talk with the patient’s baseline comorbidities and emergency clinical status.

Future research should therefore prioritize multicenter prospective and, where feasible, randomized comparative studies employing standardized clinical and neurocognitive endpoints. Such evidence, complemented by advances in cannulation, aortic occlusion devices, neuromonitoring, and intraoperative biomarker assessment, may ultimately improve outcomes and broaden surgical indications—extending them to younger patients with connective tissue disease and to high-risk patients currently deemed inoperable. Finally, we acknowledge that this is a narrative, non-systematic review and that selection bias cannot be fully excluded, despite our prioritization of higher-level evidence and our explicit labelling of the strength of evidence underlying each major claim.

## Data Availability

No new data were generated for this narrative review.*
